# Power, policy and abortion care in Uganda

**DOI:** 10.1093/heapol/czaa136

**Published:** 2020-12-21

**Authors:** Alexander Kagaha, Lenore Manderson

**Affiliations:** 1 School of Public Health, University of the Witwatersrand, Johannesburg, South Africa; 2 Institute at Brown for Environment and Society, Brown University, Providence, RI, USA; 3 Department of Anthropology, School of Social Sciences, Menzies Building 20 Chancellors Walk, Monash University, Clayton VIC 3800, Australia

**Keywords:** Uganda, abortion care, communicative material, discursive practice, prioritization, epistemic governance, governmentality

## Abstract

Unsafe abortion practices remain the major contributor to maternal death in Uganda, impeding the achievement of universal health coverage and quality of maternal health care. Using an ethnographic design and critical discourse analysis, we explored the operations of power in setting maternal healthcare priorities, as evident at the 2018 Reproductive, Maternal, Neonatal, Child and Adolescents Health Conference. Observational data were collected of the policy-making activities, processes and events and key informant interviews were conducted with 27 participants. We describe how neoliberal and state governance through the structure and organization of policy-making, epistemic governance and universal concepts of ‘high-impact’ interventions, results-based financing, cost-effectiveness and accountability converge to suppress the articulation of local conditions associated with unsafe and risky abortion. By defining maternity along the continuum of birth and emphasizing birthing women, priority-setting was directed towards interventions promoting women’s normative role as mothers while suppressing unmet abortion care needs. Finally, discursive and communicative materials controlled how women of reproductive age in Uganda managed reproduction.

**KEY MESSAGES**

- Insufficient attention has been paid to the low priority given to abortion delivery in national maternal healthcare responses.
- Neoliberal rationality actively produces compliance and self-regulation. It creates an infrastructure in which participants prioritize maternal healthcare issues with high-impact interventions.
- Normalizing metanarratives of motherhood and the fear of the disciplinary effects of biopolitics compel advocates of abortion care delivery into self-regulating conduct.
- The organizing effect, epistemic governance and production of compliant and self-regulating participants suppress opportunities to illuminate structural and institutional conditions associated with unsafe abortion care, and so undermine reforms for quality abortion care delivery.
- By examining the performative and productive ways in which neoliberalism, the state and the health institution govern policy making, we uncover how abortion care delivery discourses are silenced.

## Introduction

Maternal deaths remain a concern in both restrictive and liberalized legal contexts. Each year, globally, between 4.7% and 13.2% of maternal deaths are attributed to unsafe abortion (Say *et al.*, 2014; Ganatra *et al.*, 2017), with increasing numbers of these deaths associated with poor quality of care in health facilities (Namazzi *et al.*, 2015). For social and political reasons, comprehensive abortion care delivery is not included in national responses for maternal healthcare improvement in many African countries; Uganda is one. In this article, we consider policy making concerning maternal health, and examine how maternal healthcare issues are identified to assist in developing responsive and preventive interventions. In this context, we explain the silencing of unsafe abortion and the provision of abortion care.

In Uganda, policy-making is technically the responsibility of the Ministry of Health (MoH). However, to align national maternal health priorities to the Sustainable Development Goals, global networks like Reproductive, Maternal, Neonatal, Child and Adolescents Health (RMNCAH) have taken centre stage to support maternal healthcare programming (Ministry of Health, 2016). RMNCAH is an area of maternal healthcare programming which brings actors together into a network through which maternal healthcare issues are identified to assist in developing responsive and preventive interventions.

Maternal healthcare investment is done through the RMNCAH Stakeholders’ Conference. Stakeholder participation in policy making is assumed to be an effective means for advocacy, empowering actors to articulate neglected maternal health problems and increasing government accountability (Klugman, 2008; Smith and Shiffman, 2016). However, networks may activate intercountry and local tensions (Storeng *et al.*, 2019), and participation in policy making may result in individual participants self-restraining their discursive conduct (Lorenzini, 2018).

Maternal healthcare priority setting occurs in the context of neoliberalism. In this article, we use this term to refer to a distinct form of reason or rationality that organizes everyday life, and shapes or governs the conduct of individuals in relation to others for economically rational reasons. This encourages individuals to take specific entrepreneurial action and introduces techniques of self-regulation (Lemke, 2001). The prioritization of policies is informed by global views and the cultures that legitimate them (Alasuutari and Qadir, 2014).

Priority-setting is shaped by the demand for universal health coverage (UHC), quality of care improvement, results-based financing (RBF) and cost-effectiveness, with neoliberal market forces informing both public health and global norms of safe motherhood (Béhague and Storeng, 2013; Storeng and Be, 2016; Witter *et al.*, 2017). Neoliberal meta-narratives that privilege reproduction and motherhood shape national maternal health policy-making, while muting questions of abortion care. Normalizing narratives shape the interpretation and appropriation of policy issues (Walsh, 2007; Levinson *et al.*, 2009).

Evidence to justify policy priorities often casts an image of rationality (Parkhurst *et al.*, 2018), making interventions that are easier to measure and evaluate more attractive than structural reforms. Accordingly, technologies, knowledge production and communication shape how policy issues are understood and how priority setting occurs (Jacob and Hellström, 2018). We refer to this as a form of epistemic governance which may privilege dominant perspectives at the expense of others. Epistemic governance shapes perceptions of the world, and determines what is prioritized and used by donors to hold recipient countries accountable (Inda, 2005; Béhague and Storeng, 2013; Alasuutari and Qadir, 2014; Oren, 2017). Specific epistemic stances define what is possible regarding women’s health and maternity. In this context, policy priority setting is based on existing statistical evidence bases, with qualitative evidence undervalued. Equally, communicative relationships draw on accumulated knowledge of specific communities (Hook, 2001) and may involve struggles for control (Emmons, 2009), since knowledge bestows power to those who generate and use it (Hook, 2001; Emmons, 2009). Existing forms of knowledge, expertise and means of reasoning govern maternal healthcare priorities, which fail to address continued maternal deaths resulting from unmet abortion care needs. Specific constructions and communication of knowledge renders abortion care invisible and deprioritized.

Elsewhere, we have described how (post)abortion care (PAC) in Uganda is delivered within the constraints of restrictive state law on abortion and morality (Kagaha and Manderson, 2020). In 2015, stakeholders developed and launched national guidelines and standards to prevent maternal mortality due to unsafe abortion, but following resistance, these were withdrawn 6 months later (Cleeve *et al.*, 2016). Policy making on abortion is clouded in moral discourses of abortion as evil and immoral (Larsson *et al.*, 2015), with abortion care delivery framed by medical, legal and moral lenses and ‘normalizing judgements’ that produce compliant behaviours (Thiel, 2019). Christian morality shapes which policy priorities are conceivable and politically possible (Haaland *et al.*, 2019), affecting the legitimation processes (Korkut and Eslen-Ziya, 2016) through which global norms direct the state’s behaviours. Furthermore, as we illustrate below, communicative materials hide specific realities while making others visible (Rudrum, 2016). At the same time, discursive relationships influence maternal health priority setting and produce compliant individuals (Merlingen, 2011; Thiel, 2019). Without attending to the discursive technologies that exclude abortion care from national responses, obstetric violence against women with abortion care needs remains a significant problem.

The national RMNCAH Conference to prioritize interventions for better health outcomes in Uganda was held in August 2018. The 2-day conference, organized by the MoH with multiple sources of donor support, included district health officers and participants from local and international non-government organizations (NGOs) working in maternal, sexual and reproductive health, development agencies and donor organizations. The conference was critical to developing a plan for maternal healthcare improvement, to be anchored within the national health sector strategic development plan to accelerate national progress towards maternal healthcare improvement (MoH, 2016). Before the conference, a trend and bottleneck analysis, with critical issues identified, was conducted by the MoH, and a summary of this was distributed for discussion at the conference. Subsequently, a technical working group within the MoH consolidated the conference report into strategic ‘high-impact’ interventions for national prioritization.

In this article, we draw on ethnographic research on maternal health priority setting to investigate how abortion care discourses were effaced from the national agenda for maternal healthcare investment. We specifically ask: (1) What happens during policy-making, given the legally restrictive context in Uganda? (2) How do the discursive interactions shape opportunities to include abortion care delivery among maternal healthcare responses? (3) How do questions of expanding the quality, scope and scale of delivery of abortion care get lost? In answering these questions, we blended institutional ethnography and critical discourse analysis to examine the link between policy and legal text discourse, the discursive materials, contents and contexts (see Krzyzanowski 2011).

### The legal and moral context of abortion care delivery in Uganda

Although the Government of Uganda ratified the Maputo Protocol on the rights of women, it did so with reservation to article 14(2)C on reproductive health and abortion ‘in cases of sexual assault, incest, rape and when pregnancy endangers a mother’s mental and physical well-being’ (CEHURD, 2016, p. 23). The government has resisted supporting an enabling environment to advance the rights of women with abortion care needs. Inducing abortion is allowed only on medical grounds for the purposes of saving the life of a mother (HRAPF, 2016). No statutory instruments operationalize this provision, however, and law enforcement agencies continue to use the Penal Code Act Cap. 120, which criminalizes the procurement of abortion and aiding a woman to do so (Mulumba *et al.*, 2017). National policy responses are made in light of the law, and consequently, Uganda’s sexual and reproductive health guidelines only provide for PAC services. Eighty per cent of the Ugandan population are Christian (Larsson *et al.*, 2015), and faith-based institutions, particularly the Catholic Church, strongly influence abortion discourses. In 2016, policy reforms aimed at preventing maternal deaths and mortality due to unsafe abortion were withdrawn due to contention among stakeholders (Cleeve *et al.*, 2016; HRAPF, 2016; Mulumba *et al.*, 2017).

Restrictions impact on both health workers and women of all ages, who are harassed, intimidated, arrested, convicted and imprisoned (HRAPF, 2016). This influences the decisions of many women to self-induce or seek support in unsafe environments, with people who lack adequate skills and expertise; as a result these women are at elevated risk of perforation, sepsis and death (Aantjes *et al.*, 2018; Kagaha and Manderson, 2020). In Uganda, an estimated 54 unsafe abortions per 1000 women of reproductive age occur annually (Moore *et al.*, 2014). Legal restrictions prevent state and non-state actors from developing health system capacities to respond to abortion care needs, and this result in discrimination against women with abortion care needs other than PAC as permitted by policy (Mutua *et al.*, 2018).

## Methods

### Study population and participant selection

We draw on data generated in a study focused on policy-making practice, involving a specific set of actors. As noted above, these included the organizing institution—the MoH —with participating institutions and individuals from donor and development agencies, implementing partners, local and international NGOs, and District Health Officers. Individual participants in the study in these categories were purposively selected to explore, from their experiences in the RMNCAH Conference, how maternity and abortion care issues were shaped. Table 1 summarizes the categories of participants interviewed in this study.

**Table 1:** Categories of participants interviewed

S/N	Categories of participants	Number
01	MoH	09
02	Implementing nongovernmental organization	04
03	Professional bodies (public health experts and Uganda Obstetric and Gynaecological Association)	05
04	Religious bodies	04
05	Development Agencies	05
	Total	27

### Data collection

Non-participant observations were made at a 2-day RMNCAH Conference held from 20 to 21 August 2018, and at a 1-day conference held in December 2018. We used an observation guide, developed following the conception of policy making as an assemblage of activities, actors, processes, materials and ways of knowing (Reckwitz, 2002), but also as a governmental practice which controls, directs and produces compliant individuals (Foucault, 1977; Lorenzini, 2018). Using this guide, we observed the way the conference was organized, the setting of the venue, seating arrangements, ordering of events, communicative materials (PowerPoint visualization, audio microphones, loudspeakers, video clips at the sides of the conference hall and text materials), and how these factors combined to influence outcomes. Participant interaction in both open sessions and group sessions was observed, as were the technical terms and language used and communicative materials performed. Brief notes were taken during the conference and expanded as fieldnotes at the end of each day.

The first author conducted 27 in-depth interviews with stakeholders identified in and through the conference, selected for their roles in influencing maternal health and prioritizing abortion care interventions. The interviews explored the rationales, meanings and intentions of participants, their epistemic worldviews about maternity and abortion care, their desired policy actions, and silenced positions at the conference. The interview guide was developed following theories of policy-making processes and global discourses relating to maternal healthcare investment, and was adjusted in response to ethnographic observations.

Lastly, we reviewed legal and policy text documents which regulate abortion and abortion care delivery. International covenants and documents containing global development norms on maternal healthcare investment were reviewed. We focused also on national laws and moral discourses on abortion to identify biopolitical discourses, and explored international and local institutional rules shaping mental, behavioural and discursive choices of participants in policy-making practice. We also considered the maternal health choices deemed possible or not from the perspective of local and foreign institutions.

### Data analysis

The first phase involved identifying, processing and organizing data. As noted above, emerging issues from ethnographic observations were explored in interviews, which were audio recorded and transcribed independently. Expanded field observation notes, transcription and editing were undertaken by the first author, and were imported into NVivo software. Data were inductively coded and categorized into themes and subthemes, with text data also categorized into meta or general and specific or local discourses. The second phase involved meaning-making from the themes and subthemes from specific data sources; these included: ordering of events, epistemic technical language, selection of participants and ruling relationships and the relation of this to the low priority given to abortion care delivery.

We then focused on what the discursive materials, processes and practices at the conference performed, and how participants deployed, appropriated and negotiated power within the micro-space of policy-making. We also examined the link between discursive behaviours and global neoliberal ideology shaping maternal healthcare investment and financing. In doing so, we identified mechanisms in which neoliberalism and national biopolitics hid and silenced opportunities to include abortion care delivery on the national agenda.

## Results

In this article, we identify hidden conditions that underpin the silencing in abortion care discourse. In describing and analysing the RMNCAH Conference, we critique the normative ways of organizing as a means to order and direct policy making towards specific outcomes. We examine the discursive practices involved in policy making, the play of power and the constitution of participants into compliant subjects. We then discuss these findings in relation to the effacement of abortion care from the national agenda, and the implications of this to addressing women’s abortion care needs.

### The organization and structure of the policy making practice

Three structures emerged as shaping maternal healthcare prioritization: (1) organization of the conference, (2) epistemic governance and (3) normalizing discourses. We discuss these below.

#### Organization of the conference

The conference was organized to give priority to dominant maternity conditions and ‘high-impact’ interventions such as improved antenatal care, improved delivery and postnatal care. The conference began with rapid participatory activities through which maternal healthcare issues were identified. These constituted the main discursive material from which critical maternal health issues were discussed and enlisted for prioritization. There was recognition in the background document containing preliminary maternal healthcare priorities that teenage pregnancy and unsafe abortion disproportionately affected impoverished communities, resulting in preliminary recognition of the need for interventions to address unsafe abortion. However, abortion care was dismissed as having low measurable impact.

The location of the conference contributed to effacing abortion care from national priorities. The conference was held at the Imperial Royale Hotel, a high-class venue at which many other local and international conferences are held. Situated in an affluent suburb, the hotel has a main conference hall where the symposium was held, and other medium-sized halls for break-away sessions. The conference hall, air-conditioned with comfortable seats, offered an ambiance disconnected from the socio-cultural, political and economic contexts which underpin women’s vulnerability to unsafe abortion. This, we suggest, dissipated individual and collective capacity to argue for equitable access to and the delivery of abortion and PAC.

The selection of participants emphasized the biomedical epistemic governance over maternal health issues. Most participants were technical actors in the field of health, as described above: District Health Officers and delegates from MoH, implementing partners from international and local NGOs; and donors and development agency staff. Other technical perspectives that might link maternal health care to the broader biopolitics and social and cultural context were not included. Moreover, maternal morbidity and mortality associated with unsafe abortion involved political, socio-cultural and moral reasoning that inhibited an enabling environment to discuss abortion care delivery. Although some young people were invited, their perspectives were mediated through and limited to family planning discourses articulated in the keynote addresses: i.e. they were unable to make comments on these addresses. As the medical participants focused on medical aspects of maternity, prioritization tilted towards biomedical responses. Discussions on how restrictive biopolitics make healthcare systems unresponsive to critical abortion care needs were consequently suppressed.

The order of the keynote addresses also helped silence debates on abortion care. The opening keynote addresses set the style, framing and content of discussions. In contrast, the public health keynote address that attended to interrelated maternal health issues was presented last, at the end of the second day. The speaker of the closing keynote address articulated narratives of ‘health system strengthening’, and the need for interaction and coordination of different ‘health system’ components, including ‘human resources’, ‘health policy’ and ‘law’. He also drew attention to complementary linkages among the political, social and economic conditions upon which maternal health is contingent. This keynote address articulated local structural and institutional issues overlooked in earlier keynote addresses, but by the time of this address, some participants had already left and there was little opportunity for participants to engage with the issues. This meant too that the subject matter was rendered inconsequential to the processes that shaped maternal health priorities. Hence policy and law reform, and attention to underlying drivers of unsafe abortion, constituted neither part of the discursive contents nor its outcomes.

#### Epistemic governance

Epistemic governance refers to the mechanisms in which knowledge production and communication shape how policy issues are understood and how priority setting occurs (Jacob and Hellström, 2018). We analysed the epistemic concepts and principles that were used within the conference to define maternity priorities in terms of ‘high-impact’ interventions, using statistical data, graphical trends and maps. The keynote addresses given by the representatives of global institutions provided the paradigm that shaped the way that maternal health was problematized, measured and prioritized. Table 2 summarizes the main talking points of keynote addresses at the opening session.

**Table 2:** Epistemic concepts and statements of keynote addresses

Organization	Keynote messages
World Bank Representative	Focus should be put on high impact, cost-effective interventions and results
	Support transition to long-term sustainable domestic financing of RMNCAH and scaling up financing from both domestic and international, and private and government sources
	Smart financing to ensure evidence-based, high-impact interventions are prioritized and delivered in efficient results focused way. Prioritize interventions with strong evidence base demonstrating impact
World Health Organization (WHO) Representative	Improve Quality of Care for maternal and newborns along the continuum of pregnancy, ANC, labour, delivery and period and increase access to postpartum family planning
	Mainstream accountability at all levels by addressing capacity gaps in management, administration and overall governance
	Improve mothers’ child birth experience
	Global commitment to universal access to sexual and reproductive health care
	Scale up the evidence-based/high-impact interventions for children and maternal healthcare improvement
UNFPA Representative	Uganda’s teenage pregnancy accounting for 25%, the highest in the world. Adolescents pregnancy account for 28% of maternal mortality and contraceptive use the lowest in the region, at 35%. All these result in a high population growth of 3% per annum
	Increase the domestic financing of the health sector and improve the implementation rate and accountability for funds
	The need to address commodity stockouts at all levels including the service points
Ministerial speech	The need for investment in the health system (human resources, medicines and supplies, infrastructure development, civil registration and vital statistics, data collection and reporting for evidence-based programing)
	The need to focus on high-impact interventions realize RMNCAH indicators
	UHC to reduce unmet needs for modern contraceptives, ANC, institutional deliveries, PMTCT, comprehensive obstetric and newborn care
Uganda National Population Council representative	Demographic transition: problematized how median age at marriage, low uptake of modern contraceptives Aim to accelerate family planning, public–private partnerships, human resources for health and commodities and supplies Effective and efficient service delivery (health, education), strong legislative framework and zero corruption

As illustrated in Table 2, the agenda for maternal healthcare improvement was set on the principles of ‘evidence-base’ and impact. ‘Evidence-based’ was aligned to the statistical representation of maternal health issues. Abortion and abortion care delivery issues were not statistically represented as such data are not collected by the two relevant national statistical institutions—the MoH and the Uganda Bureau of Statistics (UBOS)—reflecting the restrictive legal and policy environment. The case for integrating abortion care delivery into a maternal health policy therefore was not visible in statistical and graphical trend analysis, so shaping what was prioritized. The conception of quality attuned to quantifiable units silenced opportunities for illuminating processes and mechanisms through which poor abortion care outcomes might emerge; consequently, abortion care discourses were suppressed using epistemic means to define evidence.

Prioritization was also steered through metanarratives characterizing maternal healthcare issues (Table 2). The representative of the World Bank emphasized the investment paradigm, and spoke of ‘investment returns’, ‘demographic dividend’ ‘results-based/performance-based financing’, cost-effectiveness, efficiency and accountability in maternal healthcare delivery. This discourse provided the framework within which quality of care was defined, quantified and evaluated to enable RBF monitoring. This meant that the focus of participants was predetermined. Yet interventions that address abortion care hinge on the policy and legal reforms whose impact cannot be presented in measurable terms since they influence practice indirectly. Moreover, the implementation of abortion care interventions in Uganda is shaped by the existing neoliberal funding regime:

*If you now go to check inside the details of many of the funders, this component [abortion care delivery] is left to us. Because many of them [non-government organisations implementing maternal healthcare responses] receive money from USAID, us who may receive money like from the Koreans, we can afford to buy technological equipment for abortion care* (in-depth interview, participant from an international development agency).

Securing funds to implement abortion care delivery interventions is further compromised by the funding guidelines from the USA, known as the Mexico City Policy. This policy prohibits NGOs receiving funding from USAID to provide abortion care services or to advocate for the liberalization of abortion laws (Mavodza *et al.*, 2019). Many organizations involved in safe abortion care delivery withdrew from providing the service because of financial constraints and the fear of losing support from USAID, the major funder. Thus, advocacy organizations with funding linked to the USAID cut their activities.

*Today, the USAID for instance, which is our biggest funder, cannot put money into procuring equipment for post abortion care because of their gag rule [Mexico City Policy]. It is not a Ugandan policy, people [implementing partner organisations] are trying to see that these people [women with abortion care needs] are being managed but the government is having no capacity. They [USAID] have withdrawn all the funding for such services* (in-depth interview, development partner).

As illustrated, the withdrawal of funding for abortion care service delivery compromised health facility capacities and staff preparedness to provide care for patients with unmet abortion care needs. Although some organizations access funding from alternative and anonymous funding under the umbrella of the ‘She decides’ movement, a global solidarity movement for gender equality, adjusting in the short term is very difficult. Moreover, although resistance against the global gag rule created the movement for alternative funding, this does not transform local biopolitics that operate through the restrictive law on abortion and an unclear policy environment, as one respondent noted:

*When they are giving you the funding, they say their activities should be only carried out in countries where abortion is legalised. So, our work is to try and change people and fight for the rights of women. So that the policy is changed but for them in their phrase they put that, if the country does not support them; they can’t give you the money. That is how we failed* (in-depth interview, participant from international implementing partner organization).

This makes it difficult to have interventions which enable abortion care, including PAC, in Uganda. Moreover, without legislative reforms and changes in the policy and normative environment, translating global commitment for UHC of maternal health services becomes difficult:

*We do a lot of dialogue meetings with ministry especially at high level, like the ambassadors and the minister of health, and other ministers as well. But I think bringing the global commitment into the policies and regulations in the country so that they [the state institutions] are following what they are committed to, is a general challenge. It is more difficult to try to get them implementing their commitments than for them to sign documents* (in-depth interview, participant from a development agency).

Metanarratives at the conference also focused on normalized maternal conditions. For instance, the ministerial statement on maternal healthcare addressed improvement along the continuum of birth (pregnancy, antenatal care, labour, delivery and postpartum). This helped attune RMNCAH participants to prioritize maternal healthcare responses along women’s expected experience of reproduction and roles as mothers, and did not allow for discussions on ex-nuptial pregnancies and births, infertility, miscarriage, stillbirth or abortion. Although abortion care services could be addressed through interventions aimed at articulating the needs of mothers, many unmarried women and adolescents also often experience abortion care needs but were excluded from discussions on the continuum of motherhood. Moreover, among married women, abortion care needs may arise outside normative roles, and these women too were neglected and their concerns were neither articulated nor prioritized. Even so, at the end of the conference, delegates called for the integration of ‘harm reduction in the implementation guidelines in PAC e.g. MVA, FP and managing complications’. By focusing on PAC, this integration ignored questions of comprehensive abortion care delivery and the conditions in which poor quality of abortion care outcomes occur at health facilities.

### The discursive practice

Discursive practice refers to the way people explain their experiences through the communicative relationships in which they are embedded (Hook, 2001; Kwon *et al.*, 2009; Wodak and Meyer, 2009; Macgilchrist and Van Hout, 2011; Clarke *et al.*, 2012). The analysis of discursive practices allows us to describe and explore the social structures and the environment through which maternity is constructed and its responses framed, and to illuminate the role of communicative materials, including policy and law. In this study, the analysis of discursive practices focused on the dominant actors—i.e. health professionals and policy makers.

#### Ruling relationships

Ruling relationships here denote the ways in which hierarchical relationships structure and control individual behavioural practices in maternal healthcare priority settings. In this study, we examined the performance of relationships at the RMNCAH Conference. Discursive relationships shaped the behaviours of participants. First, participants sought to maintain and exhibit the social image of the first lady, who was patron of maternal healthcare interventions. In follow-up interviews, advocates for safe abortion care delivery stated that the presence of participants from state institutions made it difficult to openly articulate rationales for abortion care delivery, and some people preferred to stay silent. Some participants felt that they were being watched and would be sanctioned, ostracized or labelled. This affected how abortion care was articulated during priority setting activities:

*We were not ourselves. It was an engagement, even … especially the Ministry [of Health] officials were there. I think the ministry was the biggest problem. So, we were very careful of the language we used, how we packaged these issues [abortion care]. This is how we have been there for all these years working. If it means using the wrong language that you really don’t mean, then do that. If it means calling abortion, post-abortion, then do that, so that everybody is comfortable* (in-depth interview with a respondent from an NGO).

As suggested, advocates for abortion care services carefully negotiated morality and law in their articulation. Packaging the need for safe abortion as PAC silenced any conversation around the critical issues that relate to and affect induced abortion that continues to be unsafe and risky for women of all ages. Advocates used silence as a strategy to avoid being noticed by political and regulatory institutions, and ostracized in development interventions, stigmatized and labelled abortionists. However, to avoid state surveillance and their self-regulation, their expressive agency for abortion care delivery was suppressed.

The relationships of resistance extended beyond the conference to the abortion care landscape, where advocates sought to provoke state responsibility. They worked behind development agencies to have their advocacy issues articulated, and had unsuccessfully funded a private member’s bill in parliament to bring about law reform. Where advocates pursued legitimate legal reforms, the government deployed the common discursive statement, ‘we are working on it’, without any meaningful response. In some cases, state institution representatives simply ignored public statements of the need for abortion care, which activists had designed to provoke response. For instance, research reports that articulated the vulnerability of very young women to unsafe abortion in one of the districts in south-eastern Uganda was targeted to provoke a response from both the MoH and the national legislature, but, despite media coverage and the invitation to comment to key government actors, it failed to elicit a response. Thus, silence as a tactic of power was used by both the state and activists. However, activists expressed defeat as government silence meant failure to transform the policy regime to advance abortion care delivery. To counter state control, some advocates turned to donors to advance their agenda:

*The other nice thing to do is to push it through the development partners, because Uganda is highly donor dependent. If a major donor said it, the government would listen; it would, you know, push back and say ‘donors will not tell us what to do’ but in the long run they do what they are told to do (laughter)* (Key Informant, International NGO).

Not all advocates agreed with this strategy. A number, constantly engaged with members of the donor community, while they recognized the power of development agencies, also recognized that they had to work within the national law:

*We do not have the back up from the UN bodies. You know, a UN body has the power to twist the arm of the government but let me tell you, all these UN agencies that we work with, UNFPA, UNICEF, what … will never do anything that they feel it is against the laws the country. So that is why we are hanging; we kick the ball this way, we hit a wall and we come back. The UN women for them they go fighting gender-based violence, they go fighting these FGM, but they will never be tough on any area of abortion* (in-depth interview, respondent, international NGO).

International development agencies also self-regulated to fit within the government’s law on abortion. In follow-up interviews, participants from international organizations and development agencies expressed the fear that their organizations would be expelled from the country if they contravened the national law, and they adhered to national priorities to ensure they could advance their own maternal health programmes. Fear of the consequences of not doing so successfully suppressed any efforts to include abortion care as a national maternal healthcare priority.

#### Communicative materials

Communicative materials also silenced discourses on abortion. Video clips, texts and textualized and visualized material (such as conference screen visualization) excluded abortion care while presenting other maternity issues. While unsafe abortion is a major contributing factor to maternal death, no video clips addressed this. Videos on fistula, health facility infrastructure and family planning, shown before and during the session breaks, were very effective: they captured people’s attention and fed into informal discussions during tea breaks. Video clips and visual materials of maternal health issues constituted points of reference during discursive engagements, and they reflected emergent and prioritized discourses on maternal health.

Discourses on safe abortion care delivery were hidden in plain sight. Texts on safe abortion and PAC services and products were available at the tables of specific organizations, located at the periphery of the main conference hall. Such texts contained discourses on family planning, safe abortion and PAC technologies including abortifacients (used for medical abortion). However, these discourses were not included in visual material or in discursive engagements on maternal healthcare improvements in plenary sessions. Even in break-away groups where the range of maternal health issues generated through pre-conference activities was assessed, abortion care was rarely discussed. Working groups identified, discussed and documented the maternal health issues they considered to be critical for the national response, but, although summaries were presented in the plenaries, the group sessions were constituted in afternoons and had fewer participants. Maternal health issues received more critical reflections in the main conference sessions than in the working groups.

## Discussion

Our interest was in understanding the mechanisms through which abortion needs were effectively obliterated from Uganda’s national maternal healthcare priorities. Our findings reveal that abortion care delivery was effaced through tools of governmentality including both (1) the structure and organization of the conference and (2) the discursive relationships through which maternal healthcare priorities were made. Epistemic governance constituted through neoliberal concepts of RBF, cost-effectiveness, efficiency and accountability tilted the prioritization towards identifying maternal health conditions that would advance the growing role of the private sector in maternal healthcare delivery. To effectively achieve this goal, epistemic governance relied on statistical data, graphs and charts of maternal health trends, and maps as measurement tools that constituted the evidence-base to measure and determine ‘high-impact’ interventions. No national statistical data were available on abortions or abortion care, and the collection and distribution of this data require reforms in policy, law and morality to ensure that interventions have a measurable impact. Epistemic governance also operated through normalizing biomedical concepts that defined maternal healthcare problems along the continuum of birth (pregnancy, antenatal care, labour, delivery and postpartum care). These normalizing techniques effaced abortion care discourses since abortion fell outside the continuum of birth.

During discursive interactions, neoliberal governing institutions and the MoH combined to shape maternal health priorities. The conference organizers prioritized biomedical participants, locking out non-medical perspectives which could have articulated the need to address underlying conditions for poor maternal health in general and abortion care in particular. The ordering of keynote addresses rendered the public health keynote speaker powerless to shape outcomes. State governmentality operated by silencing discourses on abortion care using vague and dismissive statements in response to (rare) questions, suggesting action to abortion care advocates but without any meaningful response. Fear of being excluded in the network of development actors, and being labelled as an abortionist, also suppressed discourse and ensured compliance and complicity in ignoring abortion as a health issue. Ministerial emphasis on birthing, safe motherhood, maternal and child health all combined to hide abortion care narratives which contradicted the expected roles of women as mothers.

Discursive practice was mediated through normalizing narratives that controlled the prioritization of maternal healthcare issues. Metanarratives of ‘women giving birth’, ‘safe motherhood’ and ‘maternal and child health’ characterized discussions in the conference. These led to preoccupation with strengthening health systems capacity to respond to the needs of birthing women. The metanarrative of ‘women giving birth’ fitted with societal expectations of women as mothers. Consequently, the unmet needs of women seeking to terminate pregnancy were absent. Discursive engagements could not deal with the everyday challenges in abortion care practice which health workers confront within a legally restrictive environment. The primary, almost exclusive, narrative of motherhood drew attention to the reproductive function of women and de-emphasized abortion care needs. Abortion practice was seen to vandalize moral, legal and spiritual norms, values and beliefs; women with abortion needs, and those caring for them, are considered transgressive of the collective norms and values society uses to control women’s reproductive health behaviours.

Neoliberalism shaped how maternal health was conceptualized, thought about and discussed. From the global development perspective, the narratives of achieving ‘demographic dividends’ characterized and led to family planning as the foremost national maternal healthcare priorities. This fitted neatly within the national response to the prevention of unsafe sex and the need for abortion, was embraced by conference participants, and matched global priorities that advance increased government expenditure on family planning supplies. During discussions, measurement of quality of care focused on the statistical strength of data, ignoring the processes and conditions that underpin quality care practice. In the context of a culture of investment impact, abortion care constituted a marginal health concern: it lacked critical representation in terms of statistical data on its magnitude, scope and scale. These neoliberal terms acted upon and produced compliant entrepreneurial actors, who were conscious of the economic incentives produced by RBF and found vested interests in following ‘high-impact’ maternal health interventions. This meant that women of reproductive age became a means towards financing health facilities. In this context, policy-making participants reflected more on the financial status of their health facilities than on the conditions affecting women of reproductive age. Women accordingly find themselves vulnerable to a biopolitics that regulated sexual and reproductive health behaviours.

Neoliberal and state governmentality produced compliant subjects, uncritical and unconscious of the local conditions and realities faced by women with abortion care needs and of institutional obstetric violence. Participative freedom also limited and regulated what people could speak about. Participants’ behaviours were governed by formal and informal ruling relationships, and an infrastructure of self-interest embedded in the neoliberal concepts that shape maternal health investment. In addition, participants engaged in self-constituting practices as a result of fear of stigmatization, labelling and ostracism. Advocates used silence as a strategy to avoid being detected, but this meant that they were complicit in not discussing abortion. Although some participants attempted to counter this by provoking state response and undertaking litigation, the state deployed subtle tools such as silencing and using discursive statements that gave hope without any tangible response.

Many scholars argue that lack of policy guidelines impedes abortion care delivery and inhibits quality improvements that could be facilitated by healthcare training (Cleeve *et al.*, 2016; Mandira *et al.*, 2016; Mutua *et al.*, 2018). Our findings deepen this analysis to illuminate how restrictive contexts limit attempts at policy reforms. We have examined power as exercised in the constitution of subjects through practices, discursive styles (Nunes and Lotta, 2019) and ordering of behaviours (Levinson, 2009). We have argued that advocates and opponents to abortion care often use economic, legal and moral, practical and ideological frames and social and political drivers (Kienzler, 2019). But here, we have also illuminated how operations shape outcome. While supporting the discursive operations of power, we acknowledge the epistemic effect of evidence-based policy-making. However, in the context of abortion care, the effect occurs through the creation of an infrastructure of self-interest to prioritize interventions that fit neoliberal interests. Thus, the productive nature of power contingent upon macro-economics and the political economy suppresses the struggle for discursive agency.

Our findings amplify Rudrum’s argument (2016) on the need to recognize and scrutinize texts as materials of power that may hide some realities while presenting others. This is critical because policy-making is text-mediated and embodies ruling relations (Bisaillon and Rankin, 2013). In this study, we add to this by highlighting the power of national governments to govern global interests using local biopolitics. Studying biopolitics as practices of governance helps to uncover how global development interventions and norms fail to enter into the political processes of national priority setting.

We challenge the development imperative of participation as a means to address marginal maternal health issues (Klugman, 2008). Rather than a solution, we see that participation serves as a political process that legitimizes the growing global influence of business norms within maternal healthcare investment (Jasmine and Porter, 2016). This is at the cost of addressing the local conditions that result in women’s vulnerability to unsafe abortions and unmet abortion care needs. For this reason, we suggest, neoliberal reforms for decades have failed to put abortion care delivery on national maternal health priorities in many sub-Saharan African countries.

We highlight the need to attend to the organizing effect of power in policy-making through ordering keynote addresses and selecting participants. Seemingly neutral activities including choice of venue constitute symbols of domination which successfully hide local conditions which, in this case study, underpin unsafe abortion and fail to acknowledge the need to prioritize abortion care delivery. We unexpectedly identified the active role of communicative materials in organizing, silencing and making present specific narratives. These findings illustrate how communicative materials constitute intricate ways of governing participants and self-governance. By triangulating ethnographic observations and in-depth interviews with critical discourse approach, we also illuminated the performative effect of the conference practice, and the mechanisms of power operating by linking micro practices in the conference to national biopolitics and global force of neoliberalism.

## Conclusion

In Uganda, policy-making is structured according to and situated within a global neoliberal ideological context that shapes maternal healthcare investment. Uganda’s moral and legal restrictions also impact on policy-making. At all levels, women’s sexual and reproductive health occurs in a political environment, operating through epistemic governance, the structure and organization of the policy conference and discursive relationships. As illustrated, neoliberal technologies govern policy-making, and may stifle abortion care delivery. In studying how debates influence the prioritization of abortion care delivery in national maternal healthcare improvement responses, there needs to be a move away from normative moralities, law and policy to consider ways in which neoliberal ideology, the state and health institutions govern the conduct of policy-makers. Normativity distracts attention from the intractable local structural and institutional violence that creates vulnerability to unsafe abortion.

## Funding

This research on which this article is based was conducted as part of the first author’s doctoral study. The research was supported by the Consortium for Advanced Research Training in Africa (CARTA). CARTA is jointly led by the African Population and Health Research Center and the University of the Witwatersrand and funded by the Carnegie Corporation of New York [B 8606.R02], Sida [54100113], the DELTAS Africa Initiative [107768/Z/15/Z] and Deutscher Akademischer Austauschdienst (DAAD). The DELTAS Africa Initiative is an independent funding scheme of the African Academy of Sciences (AAS)’s Alliance for Accelerating Excellence in Science in Africa (AESA) and supported by the New Partnership for Africa’s Development Planning and Coordinating Agency (NEPAD Agency) with funding from the Wellcome Trust (UK) and the UK government. The statements made and views expressed are solely the responsibility of the Fellow. It was also partially funded by an African Doctoral Dissertation Research Fellowship (ADDRF) award offered by the African Population and Health Research Center (APHRC) and in partnership with Ipas, Guttmacher Institute, Gynuity Health Projects and Ibis Reproductive Health.

*Conflict of interest statement*. None.

*Ethical approval.* Ethical approval was obtained for the study from the Human Research Ethics Committee (medical) of the University of the Witwatersrand (reference number M180466), the Uganda National Council of Science and Technology (UNCST) (reference number SS4677) and the College of Humanities and Social Sciences Research Ethics Committee of Makerere University (MAKSS REC 05.18.166).

## References

[czaa136-B1] Aantjes CJ , Gilmoor A, Syurina EV, Crankshaw TL. 2018. The status of provision of post abortion care services for women and girls in Eastern and Southern Africa: a systematic review. Contraception 98: 77–88. 10.1016/j.contraception.2018.03.014 29550457

[czaa136-B2] Alasuutari P , Qadir A. 2014. Epistemic governance: an approach to the politics of policy making. European Journal of Cultural and Political Sociology 1: 67–84.

[czaa136-B3] Béhague D , Storeng K. 2013. Pragmatic politics and epistemological diversity: the contested and authoritative uses of historical evidence in the Safe Motherhood Initiative. Evidence & Policy: A Journal of Research, Debate and Practice 9: 65–85.

[czaa136-B4] Bisaillon L , Rankin JM. 2013. Navigating the politics of fieldwork using Institutional Ethnography: strategies for practice. Forum: Qualitative Social Research 14: 1–27.

[czaa136-B5] CEHURD. 2016. Facing Uganda’s Law on Abortion Experiences from Women & Service Providers*.* Kampala: Centre for Health, Human Rights and Development.

[czaa136-B6] Clarke I , Kwon W, Wodak R. 2012. A context-sensitive approach to analysing talk in strategy meetings. British Journal of Management 23: 455–73.

[czaa136-B7] Cleeve A , Oguttu M, Ganatra B et al 2016. Time to act—comprehensive abortion care in East Africa. The Lancet Global Health 4: e601–2. 27539801 10.1016/S2214-109X(16)30136-X

[czaa136-B8] Emmons K. 2009 Uptake and the biomedical subject. In: Bazerman C (ed.). Genre in a Changing World. Lafayette: Parlor Press, 134–57.

[czaa136-B9] Foucault M. 1977. Security, Territory, Population: Lectures at the College De France, 1977–78. Pages 1-552. Translated by Burchell Graham Senellart M, Davidson IA (eds). Palgrave Macmillan. UK.

[czaa136-B10] Ganatra B , Gerdts C, Rossier C et al 2017. Global, regional, and subregional classification of abortions by safety. The Lancet 390: 2372–81. 10.1016/S0140-6736(17)31794-4 PMC5711001 28964589

[czaa136-B11] Haaland MESS , Haukanes H, Zulu JM et al 2019. Shaping the abortion policy—competing discourses on the Zambian termination of pregnancy act. International Journal for Equity in Health 18: 1–11. 30691459 10.1186/s12939-018-0908-8 PMC6348644

[czaa136-B12] Hook D. 2001. Discourse, knowledge, materiality, history: Foucault and discourse analysis. Theory & Psychology 11: 521–47.

[czaa136-B13] HRAPF. 2016. The Enforcement of Criminal Abortion Laws in Uganda and Its Impact on the Human Rights of Women and Health Workers. Kampala: Human Rights Awareness and Promotion Forum.

[czaa136-B14] Inda JX. 2005. Anthropologies of Modernity: Foucault. *Governmentality, and Life Politics (First; Inda JX Ed), Blackwell publishing limited, Oxford, UK.*

[czaa136-B15] Jacob M , Hellström T. 2018. Epistemic governance and the conditions for knowledge production in HER institutions. Studies in Higher Education 43: 1711–7.

[czaa136-B16] Jasmine G , Porter F. 2016. Challenging gendered inequalities in global health: dilemmas for NGOs. Development and Change 47: 782–97.

[czaa136-B17] Kagaha A , Manderson L. 2020. Medical technologies and abortion care in Eastern Uganda. Social Science & Medicine 247: 112813. 32058197 10.1016/j.socscimed.2020.112813 PMC7613281

[czaa136-B18] Kienzler H. 2019. Comment mental health in all policies in contexts of war and conflict. The Lancet Public Health 4: e547–8. 31677773 10.1016/S2468-2667(19)30208-7

[czaa136-B19] Klugman B. 2008. Advocating for abortion access: lessons and challenges. IDS Bulletin 39: 10–7.

[czaa136-B20] Korkut U , Eslen-Ziya H. 2016. The discursive governance of population politics: the evolution of a pro-birth regime in Turkey. Social Politics: International Studies in Gender, State & Society 23: 555–75.

[czaa136-B21] Krzyzanowski M. 2011. Ethnography and critical discourse analysis: towards a problem-oriented research dialogue. Critical Discourse Studies 8: 231–8.

[czaa136-B22] Kwon W , Clarke I, Wodak R. 2009. Organizational decision-making, discourse, and power: integrating across contexts and scales. Discourse & Communication 3: 273–302.

[czaa136-B23] Larsson S , Eliasson M, Allvin MK et al 2015. The discourses on induced abortion in Ugandan daily newspapers: a discourse analysis. Reproductive Health 12: 1–10. 26108479 10.1186/s12978-015-0049-0 PMC4481262

[czaa136-B24] Lemke T. 2001. ‘ The birth of bio-politics’: Michel Foucault’s lecture at the Collège de France on neo-liberal governmentality. Economy and Society 30: 190–207.

[czaa136-B25] Levinson BAU , Sutton M, Winstead T. 2009. Education policy as a practice of power: theoretical tools, ethnographic methods, democratic options. Educational Policy 23: 767–95.

[czaa136-B26] Lorenzini D. 2018. Governmentality, subjectivity, and the neoliberal form of life. Journal for Cultural Research 22: 154–66.

[czaa136-B27] Macgilchrist F , Van Hout T. 2011. Ethnographic discourse analysis and social science. Forum: Qualitative Social Research 12 (1), 1-18: 10.17169/fqs-12.1.1600

[czaa136-B28] Mandira P , Näsström BS, Klingberg-Allvin M, Kiggundu C, Larsson CE. 2016. Healthcare providers balancing norms and practice: challenges and opportunities in providing contraceptive counselling to young people in Uganda—a qualitative study. Global Health Action 9: 30283–9. 27174861 10.3402/gha.v9.30283 PMC4865766

[czaa136-B29] Mavodza C , Goldman R, Cooper B. 2019. The impacts of the global gag rule on global health: a scoping review. Global Health Research and Policy 4: 26. 31485484 10.1186/s41256-019-0113-3 PMC6714436

[czaa136-B30] Merlingen M. 2011. From governance to governmentality in CSDP: towards a Foucauldian research Agenda. Journal of Common Market Studies 49: 149–69.

[czaa136-B31] Ministry of Health. 2016. Investment Case for Reproductive, Maternal, Newborn, Child and Adolescent Health Sharpened Plan for Uganda 2016/17–2019/20. Kampala: Ministry of Health. http://health.go.ug/sites/default/files/RMNCAH Sharpened Plan%2C Final June 2017.pdf

[czaa136-B32] Moore MA , Kibombo R, Cats-Baril D. 2014. Ugandan opinion-leaders’ knowledge and perceptions of unsafe abortion. Health Policy and Planning 29: 893–901. 24064047 10.1093/heapol/czt070

[czaa136-B33] Mulumba MM , Kiggundu CC, Nassimbwa JJJ, Nakibuuka NM. 2017. Access to safe abortion in Uganda: leveraging opportunities through the harm reduction model. International Journal of Gynecology & Obstetrics 138: 231–6. 28455836 10.1002/ijgo.12190

[czaa136-B34] Mutua MM , Manderson L, Musenge E, Achia ONT. 2018. Policy, law and post-abortion care services in Kenya. PLoS One 13: e0204240. 30240408 10.1371/journal.pone.0204240 PMC6150499

[czaa136-B35] Namazzi G , Waiswa P, Nakakeeto M et al 2015. Strengthening health facilities for maternal and newborn care: experiences from rural eastern Uganda. Global Health Action 8: 24271–8. 25843496 10.3402/gha.v8.24271 PMC4385205

[czaa136-B36] Nunes J , Lotta G. 2019. Discretion, power and the reproduction of inequality in health policy implementation: practices, discursive styles and classifications of Brazil’s community health workers. Social Science & Medicine 242: 112551. 31622914 10.1016/j.socscimed.2019.112551 PMC6853157

[czaa136-B37] Oren E. 2017. Using statistics to effect the societal perception through governmentality: SAM as a method proposal. Economics World 5: 477–85.

[czaa136-B38] Parkhurst J , Ettelt S, Hawkins B. (eds). 2018. *Evidence Use in Health Policy Making: An International Public Policy Perspective*, pages 1–245. Cham, Switzerland, http://eprints.lse.ac.uk/90272/1/Parkhurst_Evidence use in health policy_2018.pdf

[czaa136-B39] Reckwitz A. 2002. The status of the “material” in theories of culture: from “social structure” to “artefacts”. Journal for the Theory of Social Behaviour 32: 195–217.

[czaa136-B40] Rudrum S. 2016. Institutional ethnography research in global south settings: the role of texts. International Journal of Qualitative Methods 15: 160940691663708.

[czaa136-B41] Say L , Chou D, Gemmill A et al 2014. Global causes of maternal death: a WHO systematic analysis. The Lancet Global Health 2: e323. 25103301 10.1016/S2214-109X(14)70227-X

[czaa136-B42] Smith SL , Shiffman J. 2016. Setting the global health agenda: the influence of advocates and ideas on political priority for maternal and newborn survival. Social Science & Medicine 166: 86–93. 27543685 10.1016/j.socscimed.2016.08.013 PMC5034850

[czaa136-B43] Storeng KT , Béhague DP. 2016. “Lives in the balance”: the politics of integration in the Partnership for Maternal. Health Policy and Planning 31: 992–1000. 27106911 10.1093/heapol/czw023 PMC5013778

[czaa136-B44] Storeng KT , Palmer J, Daire J, Kloster MO. 2019. Behind the scenes: international NGOs’ influence on reproductive health policy in Malawi and South Sudan. Global Public Health 14: 555–69. 29537338 10.1080/17441692.2018.1446545

[czaa136-B45] Thiel J. 2019. The UK national student survey: an amalgam of discipline and neo-liberal governmentality. British Educational Research Journal 45: 538–553.

[czaa136-B46] Walsh JD. 2007. A birth centre’s encounters with discourses of childbirth: how resistance led to innovation. Sociology of Health & Illness 29: 216–32. 17381814 10.1111/j.1467-9566.2007.00545.x

[czaa136-B47] Witter S , Govender V, Ravindran TKS, Yates R. 2017. Minding the gaps: health financing, universal health coverage and gender. Health Policy and Planning 32: v4–12. 28973503 10.1093/heapol/czx063 PMC5886176

[czaa136-B48] Wodak R , Meyer M. 2009. Critical discourse analysis: history, agenda, theory, and methodology. In: Wodak R, Meyer M (eds). Methods for Critical Discourse Analysis. London: Sage, pp. 1–33.

